# Efficacy of a Solution Containing 33% Trichloroacetic Acid and Hydrogen Peroxide in Decontaminating Machined vs. Sand-Blasted Acid-Etched Titanium Surfaces

**DOI:** 10.3390/jfb15010021

**Published:** 2024-01-12

**Authors:** Giacomo Baima, Federica Romano, Ilaria Roato, Alessandro Mosca Balma, Riccardo Pedraza, Maria Giulia Faga, Federico Amoroso, Clarissa Orrico, Tullio Genova, Mario Aimetti, Federico Mussano

**Affiliations:** 1 Department of Surgical Sciences, University of Turin, 10126 Turin, Italy; federica.romano@unito.it (F.R.); ilaria.roato@unito.it (I.R.); alessandro.moscabalma@unito.it (A.M.B.); riccardo.pedraza@unito.it (R.P.); federico.amoroso@polito.it (F.A.); mario.aimetti@unito.it (M.A.); federico.mussano@unito.it (F.M.); 2 DIMEAS, Politecnico di Torino, 10129 Turin, Italy; 3 Institute of Sciences and Technologies for Sustainable Energy and Mobility, National Council of Research, 10135 Turin, Italy; mariagiulia.faga@stems.cnr.it; 4 Fondazione Ricerca Molinette—Onlus, A.O.U. Città della Salute e della Scienza, 10126 Turin, Italy; clarissa.orrico@unito.it; 5 DBIOS, University of Turin, 10123 Turin, Italy; tullio.genova@unito.it

**Keywords:** decontamination, titanium surfaces, biofilm, adipose-derived mesenchymal stem cells (ASCs), peri-implant treatment

## Abstract

This in vitro study assessed the efficacy of a solution containing 33% trichloroacetic acid (CCl_3_COOH; TCA) and hydrogen peroxide (H_2_O_2_) in decontaminating machined (MAC) and sand-blasted acid-etched (SBAE) titanium surfaces. A total of 80 titanium disks were prepared (40 MAC and 40 SBAE). *Streptococcus sanguinis* and *Enterococcus faecalis* strains were incubated on 36 samples, while the remaining 44 were kept as controls. Roughness analysis and scanning electron microscopy were used to evaluate the surface features before and after TCAH_2_O_2_ treatment. The viability of human adipose-derived mesenchymal stem cells (ASCs) after TCAH_2_O_2_ decontamination was assessed with a chemiluminescent assay along with cell morphology through fluorescent staining. TCAH_2_O_2_ preserved the surface topography of MAC and SBAE specimens. It also effectively eradicated bacteria on both types of specimens without altering the surface roughness (*p* > 0.05). Also, no significant differences in protein adsorption between the pristine and TCAH_2_O_2_-treated surfaces were found (*p* = 0.71 and *p* = 0.94). While ASC proliferation remained unchanged on MAC surfaces, a decrease was observed on the decontaminated SBAE specimens at 24 and 48 h (*p* < 0.05), with no difference at 72 h (*p* > 0.05). Cell morphology showed no significant changes after 72 h on both surface types even after decontamination. This study suggests TCAH_2_O_2_ as a promising decontamination agent for titanium surfaces, with potential implications for peri-implant health and treatment outcomes.

## 1. Introduction

Dental implants have become the therapy of choice for rehabilitating edentulous patients, effectively improving their masticatory function and quality of life [1]. However, some biological complications may occur and jeopardize the long-term clinical success and survival of these widely adopted therapeutic tools [2,3,4]. Indeed, peri-implantitis is a chronic inflammatory process involving soft and hard tissue around the osseointegrated implants, leading to the formation of a peri-implant pocket with consequent bone loss [3]. The prevalence of this disease was recently estimated to be between 25% and 40% in clinical practice, varying based on the case definition adopted [2,5,6]. Despite being a multifactorial condition, the primary etiological factor associated with peri-implantitis onset and progression is the microbial plaque biofilm at the implant surface [7,8,9,10]. Other risk factors/indicators have been consistently identified, with poor plaque control, a history of periodontitis, smoking and a lack of adherence to peri-implant supportive care being the most relevant [2,11,12]. Based on its etiopathology, all treatment strategies for peri-implantitis should first aim at controlling peri-implant infection [7,13].

In order to reduce or theoretically remove biofilms from contaminated titanium surfaces, different chemical or physical methods have been proposed during the last few decades, with machined surfaces being more easily cleansable than roughened surfaces [14,15,16]. Nevertheless, chemical cleaning solutions tested so far, both alone or in combination with mechanical debridement, showed limited efficacy in completely removing bacterial biofilm [17,18]. Inconsistent results were also presented using lasers [19,20] or photodynamic therapy [21]. Interestingly, resective approaches involving titanium brushes and implantoplasty remain a preferred way to remove infected contaminants in clinical practice [22,23]. However, when the re-osseointegration of contaminated implant surfaces is intended, the quality of the implant surface after decontamination is deemed an important predictor of the future outcome [24]. Indeed, some studies have questioned the real benefits of using more aggressive mechanical methods [25,26]. It is a concern that, during the cleaning of the implant, surface contaminants and microparticles of titanium can be dispersed in the surrounding tissue [27]. It is also contested that altering the titanium oxide surface layer could compromise the future reosseointegration of the treated implant [28,29]. To this regard, chemical and air-abrasive treatments appear capable in disrupting the bacterial biofilm without significantly altering the implant surface [30,31]. However, based on the available scientific evidence, a gold-standard protocol for implant surface decontamination during both nonsurgical and surgical procedures cannot be recommended yet [7,14].

In recent years, a mixture of trichloroacetic acid (TCA; CCl_3_COOH) 33% and hydrogen peroxide (TCAH_2_O_2_), initially introduced for dermatologic applications [32,33], has also demonstrated a favorable impact for the care of oral lesions [34]. This chemical peel may trigger the growth of new epithelium and connective tissue to replace scarred or aged tissue by promoting collagen formation and the activation of growth factor synthesis, as well as increase angiogenesis [35,36]. Indeed, TCAH_2_O_2_ improved the healing of oral soft tissue wounds in a canine model through upregulating the cell growth and the viability of gingival fibroblasts, suggesting its suitability to be used in periodontal and peri-implant defects [37]. This effect may result from the combined effect of TCA as a healing catalyzer and H_2_O_2_ as an antiseptic. Indeed, locally administered H_2_O_2_ for the chemical disinfection of infected dental implants has demonstrated promising outcomes, as evidenced in both in vitro studies [38] and in vivo investigations [39]. H_2_O_2_ solutions offer several advantages over alternative chemical agents, including their broad spectrum of activity against various pathogens without altering the metallurgical properties of titanium or the soft tissue [40]. The bactericidal action of H_2_O_2_ is attributed to its ability to oxidize various cellular components by virtue of its intrinsic oxidative potential and the subsequent generation of free radicals [41].

In virtue of its combined chemical properties and due to the lack of previous data, there is the rationale to test TCAH_2_O_2_ as a method to improve the infective/inflammatory conditions around dental implants. However, this potential needs to be preliminarily confirmed using in vitro studies. First and foremost, TCAH_2_O_2_ should demonstrate the ability to decontaminate the implant surface without the side effect of altering it. Therefore, the aim of this research was to assess the efficacy of the TCAH_2_O_2_ protocol in the disinfection of both smooth and roughened dental implant surfaces previously contaminated with *Streptococcus sanguinis* (*S. sanguinis*) and *Enterococcus faecalis* (*E. faecalis*) strains. These species were selected based on their robustness and prevalence in the peri-implant submucosal environment, representing a robustly utilized model to study the effect of decontaminating agents on titanium surfaces [42,43,44]. The viability and morphology of adipose-derived mesenchymal stem cells (ASCs) after TCAH_2_O_2_ decontamination were assessed as an accepted proxy of biological performance, whereas complementary tests included a protein adsorption test, field emission scanning electron microscope (FESEM) and roughness analyses.

## 2. Materials and Methods

### 2.1. Ti Disk Preparation and Study Design

**Figure 1:**
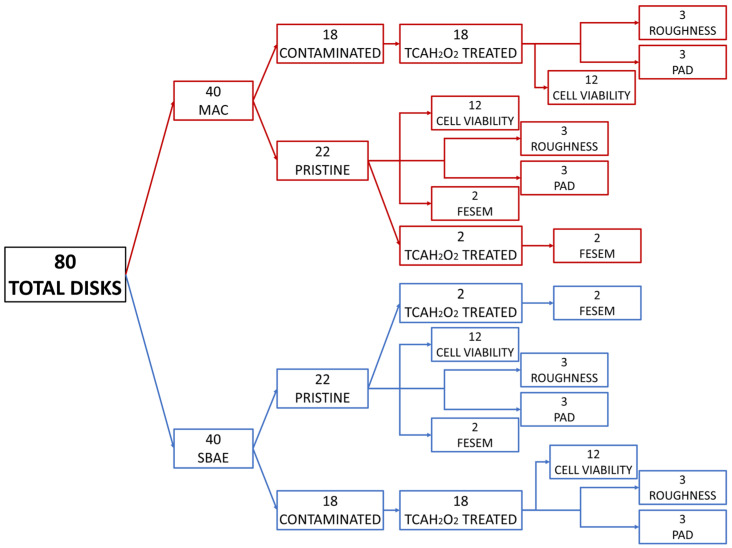
Flowchart of the experimental design. MAC, machined (red square); SBAE, sand-blasted acid-etched (blue square); TCAH_2_O_2_, trichloroacetic acid; PAD, protein adsorption; FESEM, field emission scanning electron microscopy.

For the experiments, two types of implant surfaces were prepared on titanium disks (Titanmed Srl, Lecco, Italy): the prototypical smooth control known as machined (MAC) and the widely diffused roughened sand-blasted acid-etched (SBAE). SBAE were obtained through (a) blast with alumina particles (size range 250–400 μm) and (b) immersion in hydrofluoric acid and hydrochloric/sulfuric acid mixtures. Afterward, the specimens were cleaned with sequential passages in an acetone ultrasonic bath (10 min), isopropanol (10 min), deionized water (10 min) and dried in nitrogen gas. A total of 80 disks (40 MAC and 40 SBAE) were used after the sterilization procedure, which consists of washing the samples in PBS to remove any residues and then immersing them in 96% ethanol for 20 min. After the ethanol phase, the samples were taken under a biological hood, withdrawn from the ethanol and washed once more in sterile PBS before being dried in a sterile environment inside a Petri dish. Twenty-two MAC disks and 22 SBAE disks were left uncontaminated (untreated), while two bacterial strains were grown on the remaining 36 samples, which were decontaminated through TCAH_2_O_2_ treatment (Fyox, FYOX Srl, Trieste, Italy) for 30 s to remove bacteria from the surface, and afterward thoroughly washed in a physiological saline solution, following the manufacturer’s instruction. TCAH_2_O_2_ is a chemical peeling treatment containing a mixture of TCA at 33% and H_2_O_2_ at 3% supplied in a fluid form. Both contaminated and pristine specimens underwent roughness measurement (12 samples), protein adsorption (12 samples) and cell culturing assays (48 samples). SEM analysis was performed on the pristine and TCA-treated samples (8 samples). The flowchart of the experiment is depicted in Figure 1.

### 2.2. Surface Roughness Analysis

A noncontact 3D surface profiler (Talysurf CCI 3000; Taylor Hobson Limited, Leicester, UK) was used to measure the surface roughness of the MAC and SBAE samples. Five measurements were conducted for each disk according to three amplitude parameters: Sa, Ssk and Sku. As reported in a previous study, “Sa is the arithmetic mean of the absolute values of the surface point departures from the mean plane within the sampling area. Ssk represents the deviation from the average baseline, where positive Ssk indicates a majority of peaks on the surface and negative Ssk indicates a majority of valleys. Sku describes the probability density sharpness of the profile. For surfaces endowed with low peaks and low valleys, Sku is <3; instead, it becomes >3 for surfaces with high peaks and low valleys” [45]. A Gaussian filter (cutoff value = 0.8 mm) was used to filter the surface profiles to calculate the roughness values.

### 2.3. Protein Adsorption

To quantify the protein adsorbed on the MAC and SBAE surfaces, the samples were incubated in the presence of fetal bovine serum (FBS) (2% in phosphate buffered saline (PBS)) at 37 °C for 30 min, and then they were washed twice with PBS. As described elsewhere [46], the total protein amount was first eluted from the samples with Tris Triton buffer (10 mM Tris (pH 7.4), 100 mM NaCl, 1 mM EDTA, 1 mM EG-TA, 1% Triton X-100, 10% glycerol and 0.1% SDS) for 10 min, then quantified using a Pierce™ BCA Protein Assay Kit (Life Technologies, Carlsbad, CA, USA) according to the manufacturer’s instructions using the spectrophotometer “Jenway 6300” (Jenway, London, UK).

### 2.4. Bacterial Biofilm Evaluation

Bacteria were grown overnight in 10 mL of Mueller Hinton (MH) broth (Sigma-Aldrich, Milan, Italy) at 37 °C. The day after, bacteria were subcultured until a spectrophotometric density of 0.6 at 600 nm was reached, corresponding to 1 × 10^8^ colony-forming units (CFU)/mL, approximately. Titanium samples were then colonized using *S. sanguinis* and *E. faecalis*. In particular, each disk was incubated with 1 mL of bacterial suspension in a 24-well plate using a shaking rotator (80 rpm) at 37 °C for 24 h. Then, the samples were treated or not with TCAH_2_O_2_. Then, to remove nonadherent bacteria, each disk was rinsed in sterile saline and vortexed for 10 s, six times. Disks were then transferred into a sterile plastic container with 1 mL saline solution and sonicated 3 times at 80 kHz with a power output of 150 W for 30 s [47]. Afterward, 10-fold dilutions of each supernatant were incubated in a Mueller–Hinton agar plate (Thermo Fisher Scientific™, Waltham, MA, USA) for colonies counting [48].

### 2.5. FESEM Analysis

The surface morphology of both SBAE and MAC disks, pristine and treated with TCAH_2_O_2_, was investigated using field emission scanning electron microscopy (FESEM) using a TESCAN S9000G (TESCAN GROUP, Brno, Czech Republic) to achieve a thorough visual assessment. For each disk, eight photos in In-Beam SE configuration, at a potential of 5 keV in high vacuum (0.10 Pa) and at a fixed magnification (100 k×), were taken to describe the surface morphology of the disks in order to compare the pristine surfaces with the treated ones.

### 2.6. Cell Culture and Viability Assay

Adipose stem cells ASC52telo (ASCs), hTERT immortalized adipose-derived mesenchymal stem cells (ATCC^®^ SCRC-4000, Manassas, VA, USA) cultured according to ATCC protocols, were expanded in a Mesenchymal Stem Cell Basal Medium (ATCC PCS-500-030) with a Mesenchymal Stem Cell Growth Kit (ATCC PCS-500-040). Immediately after the decontamination procedures, ASCs were seeded onto the top of the disks. Before cell seeding, a proper amount of medium was placed in each microplate well containing the samples. Then, the cell suspension, adjusted to 2.5 × 10^4^ cells/mL, was pipetted in a meandering pattern above the prepared specimen. The cells were cultured in the ASCs’ medium without antibiotics (to allow concomitant biofilm regrowth) at 37 °C in a humidified atmosphere with 5% CO_2_ for 24, 48 and 72 h. Cell Titer GLO (Promega, Madison, WI, USA) was utilized to measure the ATP release, which is associated to the viability of the cells at 24, 48 and 72 h.

### 2.7. Cell Attachment Assay

For the analysis of cell morphology, 1 × 10^4^ ASCs were seeded on the disks and cultured for 72 h; then the specimens were washed in PBS, before fixing the cells with 4% paraformaldehyde in PBS for 8 min. After being rinsed with PBS, cells were permeabilized with TBS 0.1% Triton X-100 (Sigma-Aldrich, Milan, Italy) and stained with Alexa 488-Phalloidin (Life Technologies, Milan, Italy) to detect the cytoskeleton. Images were acquired with a Nikon Eclipse Ti-E microscope using a Nikon Plan 10×/0.10 objective (Nikon Instruments, Amsterdam, the Netherlands).

### 2.8. Statistical Analysis

Descriptive statistics were presented using mean ± standard deviation (SD) and median ± interquartile range (IQR). The Gaussian distribution of quantitative data was verified using the Shapiro–Wilk test, and the one-way ANOVA or Kruskal–Wallis test was applied for parametric and nonparametric data, respectively. The level of significance was set at 0.05. Statistical analysis was performed using StataSE 17 software (StataCorp LLC, Lakeway Drive College Station, TX, USA).

## 3. Results

### 3.1. FESEM Analysis

**Figure 2:**
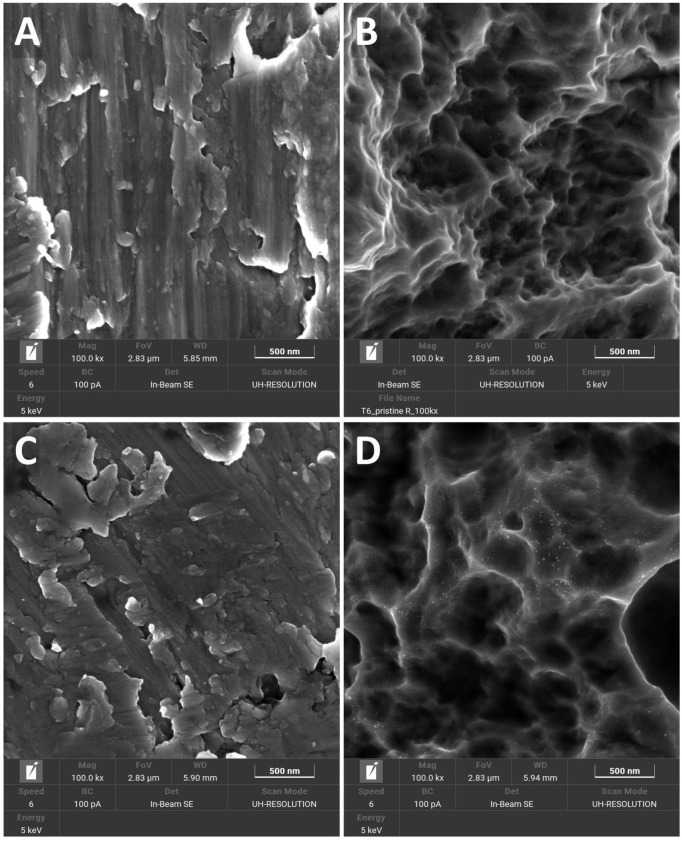
FESEM images showing the surface topography of the titanium samples at high magnification (100 k×): MAC (**A**); SBAE (**B**); MAC treated with TCAH_2_O_2_ (**C**); SBAE treated with TCAH_2_O_2_ (**D**).

FESEM analysis performed on the titanium disks revealed, for the MAC specimens (Figure 2A,C), the expected flat surface topography resulting from milling, while the typical rough pattern generated using subtractive modification techniques was visible in the SBAE samples (Figure 2B,D). No difference was apparent between the pristine (Figure 2A,B) and TCAH_2_O_2_ treated (Figure 2C,D) disks.

### 3.2. Surface Roughness Analysis

As summarized in Table 1, the data demonstrated that there were no significant alterations in roughness parameters following the application of TCAH_2_O_2_. The one-way ANOVA confirmed that there were no statistically relevant differences between the pristine and TCAH_2_O_2_-treated samples for all three parameters (Sa, Ssk and Sku) in both MAC and SBAE disks (*p* > 0.05). Also, the decontaminated group’s roughness values did not differ from the previous two in a statistically relevant way for all three parameters (Sa, Ssk and Sku) (*p* > 0.05).

### 3.3. Evaluation of Bacterial Biofilm

**Figure 3:**
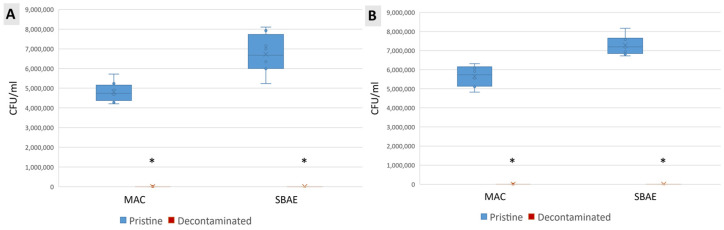
Biofilm quantification of *S. sanguinis* (**A**) and *E. faecalis* (**B**) strains on titanium samples. Data are displayed in a box plot as median ± IQR. Statistical significance was found with the one-way ANOVA test after the Shapiro–Wilk assessment for normality (α = 0.05) (* in the charts is for *p* < 0.05).

*S. sanguinis* and *E. faecalis* colonies were quantified before and after the decontamination with TCAH_2_O_2_ as reported in Figure 3. No viable bacteria could be detected after the treatment on both MAC and SBAE specimens. The groups were tested for normality, and the one-way ANOVA test revealed an immediately apparent statistically significant difference for both MAC and SBAE between the contaminated and decontaminated disks.

### 3.4. Biological Response Evaluation

**Figure 4:**
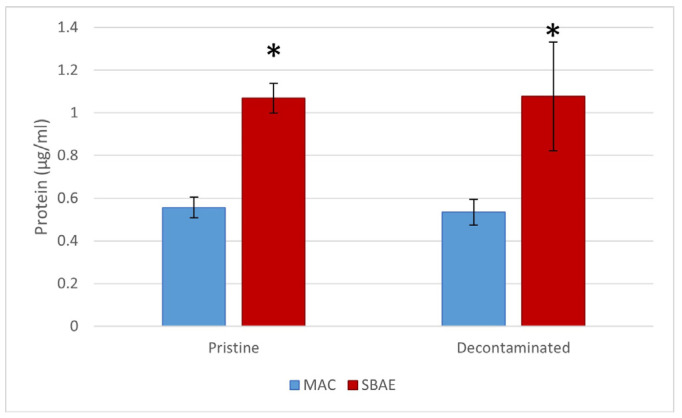
Protein adsorption. Quantification of FBS adsorbed on the pristine and decontaminated MAC and SBAE. Data are displayed as mean ± SD and refer to three independent experiments. No statistical significance was achieved between the pristine and decontaminated surfaces, while a statistical relevant difference was found between the MAC and SBAE surfaces for both the pristine and decontaminated samples (*p* < 0.05) (* in the chart is for *p* < 0.05).

Protein adsorption at the interface of a given biomaterial is correlated with the cellular response thereby elicited, which appears mandatory to ensure osseointegration. Hence, a protein adsorption assay was performed to evaluate any possible effect of TCAH_2_O_2_ at the surface of the titanium disks. As shown in Figure 4, no significant difference was detected between the pristine and TCAH_2_O_2_-treated samples for both MAC and SBAE surfaces.

**Figure 5:**
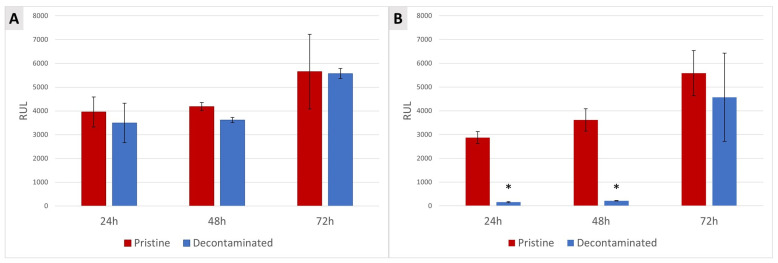
Quantification of cell proliferation assay performed on MAC (**A**) and SBAE (**B**). Data are displayed as mean ± SD and refer to four independent experiments. Statistical significance found in SBAE between the pristine and decontaminated surfaces at 24 h and 48 h (one-way ANOVA, *p* < 0.05) (* in the charts is for *p* < 0.05).

The use of TCAH_2_O_2_ did not determine any effect on ASC proliferation at any of the three time points after seeding on MAC surfaces as there was no difference between the treated and untreated MAC disks. It was instead possible to appreciate a significant decrease in cell proliferation on the TCA-cleaned SBAE samples compared to the pristine ones at 24 and 36 h, but not at 72 h (Figure 5).

**Figure 6:**
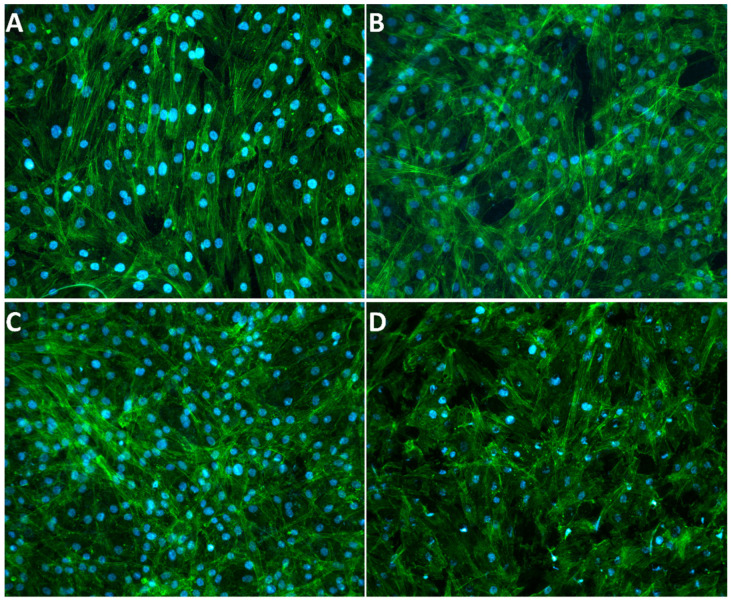
Fluorescent stain of ASCs grown, respectively, on pristine MAC (**A**), pristine SBAE (**B**), cleaned MAC (**C**) and cleaned SBAE (**D**). Actin and cell nuclei are marked, respectively, in green (Alexa 488-Phalloidin) and blue (DAPI).

Finally, to assess if and how TCAH_2_O_2_ treatment affects surface/cell interaction, in terms of cell morphology, ASCs were visualized as recurring to fluorescent staining via marking their cytoskeleton and nuclei. As shown in Figure 6, after 72 h, no evident differences between the pristine and cleaned surfaces were observed on both MAC and SBAE samples.

## 4. Discussion

The aim of this in vitro study was to evaluate the efficacy of a TCAH_2_O_2_ protocol in disinfecting both MAC and roughened dental implant surfaces that had been previously contaminated with two dental biofilm-associated bacterial strains. This study involved a comprehensive analysis of the effects of TCAH_2_O_2_ treatment on both the surface topography and the biological response of these dental implant materials. Overall, exposure to TCAH_2_O_2_ proved to be a valid decontamination method without altering topographic appearance and roughness values and while preserving biological properties.

The treatment goal when dealing with peri-implant diseases is to halt the inflammatory process and possibly favor reosseointegration, with the aim of providing long-term stable results [7]. Since peri-implantitis has a bacterial etiology, a thorough biofilm removal from the contaminated surface is pivotal to achieve this goal [3,49]. Different physical and chemical agents in diverse experimental models have been tested so far, each one presenting advantages and limitations [15,50]. In our study, to assess the effectiveness of the TCAH_2_O_2_ treatment in eliminating bacteria, representative strains of *S. sanguinis* and *E. faecalis* were incubated on the different samples. Although not mimicking all the characteristics of a mature peri-implant biofilm, the combination of these two strains was chosen because they have been consistently linked to persistent infections of human implantable devices and also to peri-implantitis lesions [44,51,52]. Notably, despite *S. sanguinis* not being a proper causative agent of peri-implant diseases, it is recognized as a key early colonizer of artificial biomaterials leading to the aggregation of more pathogenic species [43,44]. The quantification of bacterial colonies before and after TCAH_2_O_2_ treatment showed that no viable bacteria could be detected on either MAC or SBAE specimens after treatment, resembling the pristine surfaces. This highlights the robust disinfection capabilities of the TCAH_2_O_2_ protocol against the tested bacterial strains. In recent years, the antimicrobial properties of various agents, including citric acid, chlorhexidine and H_2_O_2_, have been extensively investigated. Citric acid has demonstrated effectiveness against single- and multispecies biofilms on titanium surfaces [53]. However, it has not been previously evaluated against mature biofilms and often does not surpass the efficacy of saline rinses. Chlorhexidine has exhibited bactericidal effects against both early and mature biofilms but lacks inherent cleaning properties [39,54]. Moreover, its effect as a decontaminant has been questioned by clinical studies not demonstrating any adjunctive values to its use [14,16]. H_2_O_2_ demonstrated a moderate-to-good bactericidal effect but did not exhibit obvious cleaning properties when used alone [39,55]. In our protocol, the combined solution of TCA and H_2_O_2_ was hypothesized to overcome these limitations. Notably, when other studies assessed the effectiveness of other chemical decontaminants, a complete elimination of the biofilm could not be achieved after a single application in the majority of cases, and repeated administration was needed [56,57]. Even though it was not possible to weigh the relative contribution of the two components, the biofilm was thoroughly removed after one single application of TCAH_2_O_2_ in our protocol, yielding a high interest for its transability to the clinical setting.

When reosseointegration is the goal of peri-implantitis treatment, pristine implant surface characteristics should be preserved using the physical/chemical decontaminant agent proposed [28]. In our investigation, roughness parameters remained largely unchanged after the TCAH_2_O_2_ treatment (even in the absence of previous bacterial contamination), suggesting that TCAH_2_O_2_ treatment did not significantly alter the surface characteristics of the dental implant materials. When other chemical decontaminants were applied in similar protocols, citric acid or N-acetyl-L-cysteine gave comparable results in terms of biofilm removal, although they also displayed a marked cytotoxicity on human cells [58]. We tested a novel formulation of TCA, which is widely used for dermatologic applications and has now been proposed in the dental field in virtue of its exfoliating and rejuvenating properties [59]. In our formulation, TCA was mixed with H_2_O_2_, recognized as a highly effective decontamination technique owing to its potent oxidation capabilities [60]. During this decomposition reaction, a release of highly reactive oxygen species (ROS) takes place, enabling the elimination of a wide range of organic and inorganic substances. Interestingly, when H_2_O_2_ was used alone at a relatively high concentration, tribocorrosion of titanium surfaces was observed [61,62]. Interestingly, we observed that TCAH_2_O_2_ action could maintain its decontamination properties without side effects.

The maintenance of the original implant surface characteristics is a prerequisite for the decontaminant protocol also in terms of post-wound healing cell adherence [14]. To evaluate the potential impact of TCAH_2_O_2_ treatment on the biological response of dental implant materials, several parameters were analyzed in our analysis. First, the results of the protein adsorption assay indicated no significant difference between the pristine and TCAH_2_O_2_-treated surfaces for both MAC and SBAE materials. Protein adsorption at the biomaterial interface is considered crucial for eliciting a cellular response that promotes osseointegration [63]. This suggests that the TCAH_2_O_2_ treatment did not interfere with the protein adsorption process that is critical for the integration of dental implants. Notably, TCA has a long-known effect as a protein-precipitating agent; whereas H_2_O_2_ can lead to a higher protein absorption of modified titanium surfaces, yielding higher wettability [64]. We speculate that these emergent properties of TCAH_2_O_2_ may biologically favor reosseointegration.

Second, ASCs were selected as mesenchymal precursors of osteoblasts and were seeded and grown on the titanium disks after decontamination to perform a cell viability assay (Figure 2). Despite MSCs from the bone marrow being the most well-characterized cell sources for bone regeneration, some studies indicate that there is no obvious distinction between the different MSCs harvested from the oral cavity in terms of regenerative potential [65]. The higher proliferation of ASCs on SLA than on MAC is in accordance with the literature, where the surface topography is considered a key factor for cell adhesion, and rough surfaces are preferred to smooth ones [56,66]. TCAH_2_O_2_ treatment did not affect ASC proliferation on MAC surfaces at any of the three time points studied. However, on SBAE surfaces, a significant decrease in cell proliferation was observed at 24 and 36 h post-seeding compared to the pristine surfaces. Notably, this effect was not evident at the 72 h time point. This suggests that TCAH_2_O_2_ treatment may have a transient surface-specific effect on cell proliferation, potentially connected to surface property changes, whose clinical relevance still needs to be evaluated. At a mere hypothetical level, it is conceivable that SBAE, due to its roughness, retained very small quantities of TCAH_2_O_2_, which were possibly reduced along time, allowing for the satisfactory proliferation seen at 72 h. It has to be considered that TCA has long been suggested as a means to remove the cementoclastic fibrovascular tissue associated with external root resorption lacunae, in virtue of its effect as a cytostatic agent [67]. Eventually, we aimed at assessing the impact of TCAH_2_O_2_ on cell morphology. Indeed, cell morphology can be used as a proxy for their viability and functionality. The visualization of ASCs using fluorescent staining to mark their cytoskeleton and nuclei revealed no significant differences in cell morphology between the pristine and cleaned surfaces after 72 h for both MAC and SBAE samples.

This is the first study to test in vitro the decontaminating capabilities of TCAH_2_O_2_ for dental implant surfaces. TCAH_2_O_2_ worked well even after a single application, a limit that was usually observed for other chemical decontamination products. However, this study was conducted in vitro, raising cautious interpretation of the clinical implications. The use of ASCs may not fully replicate in clinical conditions, and the transient effect on cell proliferation warrants further investigation. Moreover, the artificially produced biofilm and the aerobic culture conditions may not adequately mimic the clinical situation in terms of bacterial adherence to the underlying titanium surface. Lastly, roughness values and viability tests can only be considered as indirect proxies of the cleaning properties of TCAH_2_O_2_. Comparative studies with other disinfection methods, implant surface properties, biofilm dissolution evaluations [68,69] as well as clinical trials are needed to validate our results in practical dental implant scenarios.

## 5. Conclusions

Within the limitations of the present study, the chemical cleaning of titanium surfaces with TCAH_2_O_2_ was effective in removing bacterial biofilm from nonmodified and modified titanium surfaces and in restoring cytocompatibility. In particular, these outcomes were achieved without significantly altering the surface topography or protein adsorption. While TCAH_2_O_2_ treatment may temporarily affect cell proliferation on certain surfaces, this effect on cell morphology was transitory. These findings support the potential clinical utility of TCAH_2_O_2_ in enhancing the biocompatibility and safety of dental implant materials. Further research is warranted to investigate the long-term effects of TCAH_2_O_2_ treatment and its applicability in a clinical setting.

## Figures and Tables

**Table 1:** Surface roughness analysis of the pristine and decontaminated titanium disks (all values are expressed in µm as mean ± standard deviation).

	Pristine			TCAH_2_O_2_-Treated			Decontaminated		
	Sa	Ssk	Sku	Sa	Ssk	Sku	Sa	Ssk	Sku
MAC	0.45 ± 0.05	−0.24 ± 0.12	2.93 ± 0.15	0.44 ± 0.08	−0.26 ± 0.14	2.80 ± 0.15	0.45 ± 0.11	−0.27 ± 0.14	2.73 ± 0.13
SBAE	1.17 ± 0.07	−0.21 ± 0.53	3.26 ± 0.35	1.17 ± 0.07	−0.22 ± 0.55	3.29 ± 0.37	1.19 ± 0.07	−0.22 ± 0.58	3.17 ± 0.61

## Data Availability

Data are contained within the article.
